# Configuring intracortical microelectrode arrays and stimulus parameters to minimize neuron loss during prolonged intracortical electrical stimulation

**DOI:** 10.1016/j.brs.2021.10.385

**Published:** 2021-10-20

**Authors:** Douglas McCreery, Martin Han, Victor Pikov, Carol Miller

**Affiliations:** a Huntington Medical Research Institutes, 686 South Fair Oaks Ave, Pasadena, CA, 91105, USA; b Dept. of Biomedical Engineering, The University of Connecticut, Storrs, CT, USA; c Medipace, Inc. Pasadena, CA, USA; d Keck School of Medicine of the University of Southern California, Los Angeles, CA, USA

**Keywords:** Intracortical microelectrodes, Neural loss during electrical stimulation, Stimulus charge density, Stimulus charge per phase

## Abstract

**Background:**

Previous studies have shown that neurons of the cerebral cortex can be injured by implantation of, and stimulation with, implanted microelectrodes.

**Objectives:**

Objective 1 was to determine parameters of microstimulation delivered through multisite intracortical microelectrode arrays that will activate neurons of the feline cerebral cortex without causing loss of neurons.

**Objective:**

2 was to determine if the stimulus parameters that induced loss of cortical neurons differed for all cortical neurons vs. the subset of inhibitory neurons expressing parvalbumin.

**Methods:**

The intracortical microstimulation was applied for 7 h/day for 20 days (140 h). Microelectrode site areas were 2000 and 4000 μm^2^, Q was 2–8 nanocoulombs (nC) at 50 Hz, and QD was 50–400 μcoulombs/cm^2^.

**Results:**

Neuron loss due to stimulation was minimal at Q = 2 Ncp, but at 8 Ncp, 20%–50% of neurons within 250 μm of the stimulated microelectrodes were lost, compared to unstimulated microelectrodes. Loss was greatest in tissue facing electrode sites. Stimulation-induced loss was similar for neurons labeled for NeuN and for inhibitory neurons expressing parvalbumin. Correlation between neuron loss and QD was not significant.

Electrodes in the medullary pyramidal tract recorded neuronal activity evoked by stimulation in the cerebral cortex. The pyramidal neurons were activated by intracortical stimulation of 2 nC/phase. 140 h of microstimulation at 2 nC/phase and 50 Hz induced minimal neuron loss.

## 1. Introduction

Objective 1 was to determine parameters of microstimulation delivered through multisite intracortical microelectrode arrays that will activate neurons of the feline cerebral cortex without causing loss of neurons. Objective 2 was to determine if the stimulus parameters that induced loss of cortical neurons differed for all cortical neurons vs. the subset of inhibitory neurons expressing parvalbumin. The size of that correspondence provides an indication of the extent to which findings for all cortical neurons can be generalized to neurons which serve various functions.

Functional electrical stimulation for neuro-prostheses and for therapeutic neural modulation has assumed an important role in clinical medicine. Many of the applications now under development, including cortical-level visual prostheses [1], auditory prostheses [2,3], and intraspinal microstimulation for bladder control [4], require the high spatial resolution that is intrinsic to intraparenchymal microstimulation. For example, microstimulation in the sensorimotor cortex could provide sensory and proprioceptive feedback for closed-loop control of prosthetic limbs [2,5,6].

The “macroelectrodes” now used widely in clinical medicine have exposed areas of a few mm^2^, and the relations between neural damage induced by the stimulus charge per phase (Q) and stimulus charge density (QD) have been well investigated [7-9]. In the present study, we determined the interrelation of Q, QD and neural loss during prolonged intracortical microstimulation with multisite silicon substrate microelectrode arrays. In a previous study we determined that prolonged microstimulation in the cerebral cortex with activated iridium microwire microelectrodes (8 h/day × 30 days) with Q = 4 nC/phase, QD = ~200 μC/cm^2^ and a pulse rate of 50 Hz, the stimulations produced neuron loss within ~150 μm of the electrode tips [10]. Intraparenchymal microelectrodes with smaller electrode sites allow smaller probe shanks and supporting structures that can be implanted with less tissue injury and greater spatial resolution of the stimulus. However, the greater charge density and current density close to small electrodes may increase the risk of neuronal injury by mechanisms such as membrane electroporation [11,12]. One study using microwire electrodes implanted in the feline cerebral cortex showed stronger correlation of neural damage with Q than with QD [8]. In that study we did not evaluate differences in neuron loss while varying Q and QD independently.

It is also important to determine if the stimulus parameters that induce minimal loss of neurons are similar for different types of cortical neurons. If the excitatory and inhibitory neurons differ in their vulnerability to injury by the prolonged intracortical stimulation, the excitatory/inhibitory (E/I) balance in the cerebral cortex could be altered [13]. For example, reduction of the density of inhibitory cortical neurons has been observed in the CNS of persons with schizophrenia [13,14]. Animal models with schizophrenia-like phenotypes had fewer parvalbumin interneurons in their hippocampus [15,16].

## 2. Techniques and methods

### 2.1. Electrodes and surgical procedures

**Fig. 1.**
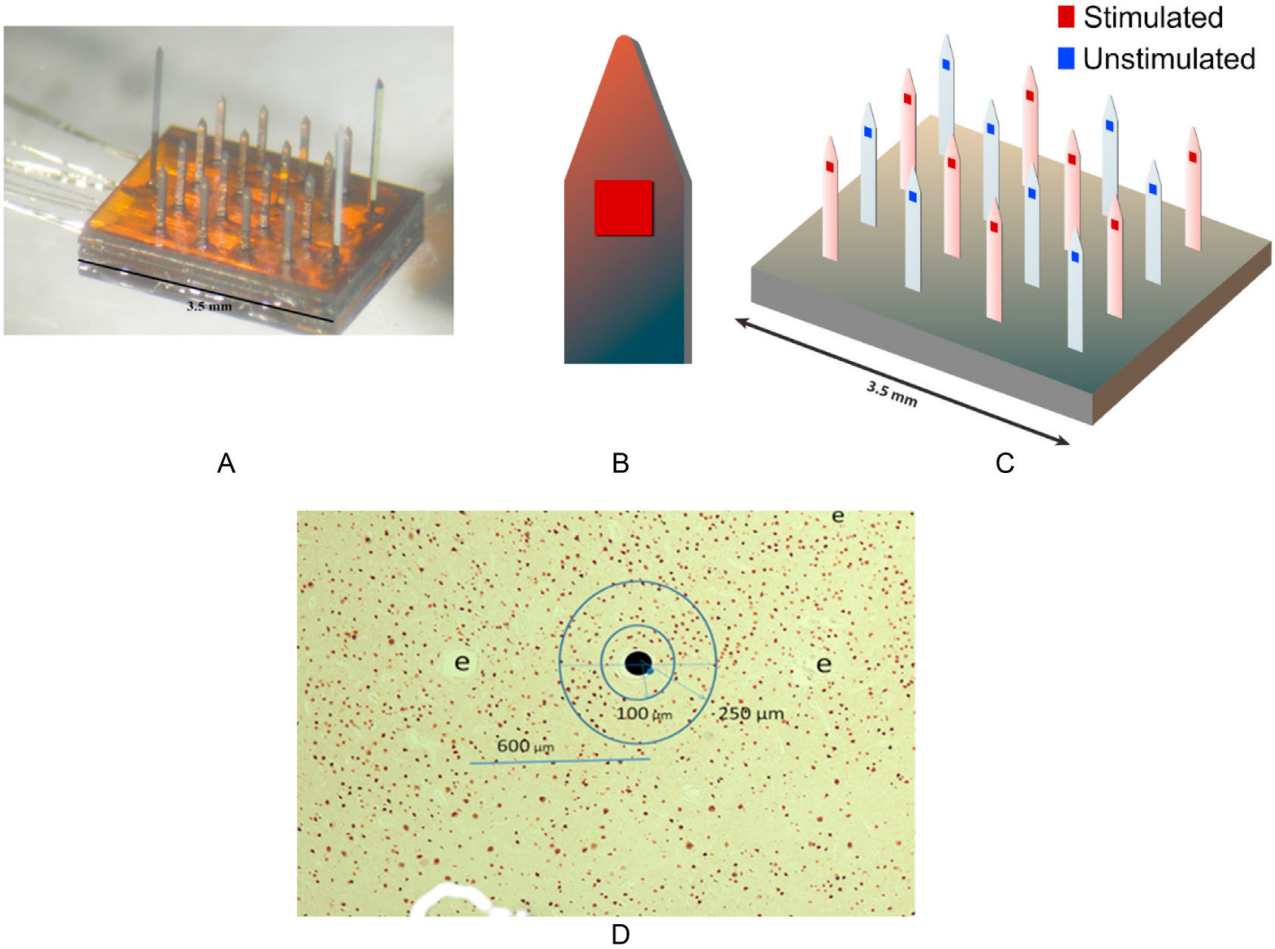
(A): Photograph of an intracortical array. The 16 microelectrode shanks, each 1.4 mm in length, are flanked by 3 longer shanks that help to stabilize the array in the cerebral cortex. (B): The tip region of a microelectrode shank showing the location of the square electrode site positioned just above the shank's tapered region above the tip. (C): The distribution of the 8 stimulated and 8 unstimulated microelectrode sites of each array. (D): Micrograph showing neurons immuno-labeled for NeuN (the small brown spots surrounding the large black spot marking the center of a microelectrode site.) Three adjacent microelectrode sites are labeled “e”. The 2 concentric annuli around the labeled electrode site are the outer boundaries of the 100 μm and 250 μm annuli within which the neurons were counted.

The procedures for implanting the intracortical arrays and for the animal's care were approved by the Huntington Medical Research Institutes' Institutional Animal Care and Use Committee (IACUC) in accordance with the National Institutes of Health Guide to the Care and Use of Animals. The study employed 15 intracortical microelectrode arrays, each with 16 penetrating microelectrodes (Fig. 1A-C) implanted into the post-cruciate cerebral cortex of 15 young adult cats. On each microelectrode shank, the single electrode site coated with activated iridium oxide [17] was located immediately above where the microelectrode shank begins to taper towards the tip (Fig. 1B), allowing identification of the histology tissue sections transecting the electrode sites. The microelectrode shanks (Fig. 1A and C) were 1.4 mm in length. In each array the rows and columns of microelectrodes were spaced 600 μm apart. In separate arrays, the geometric area of the square activated iridium oxide electrode site was either 2000 μm^2^ (7 arrays) or 4000 μm^2^ (8 array) allowing the charge density and charge per phase to be varied independently. The cat was anesthetized, and the cranium was removed over the post-cruciate gyrus of the cerebrum. The dura mater was opened and the electrode array (Fig. 1A) inserted into the cortex at a speed of 4–5 m/sec using an inserter tool of our own design [18]. The dura was sutured and the craniectomy sealed with acrylic bone cement. Bipolar recording electrodes were implanted into the medullary pyramidal tract of 8 animals. To reduce the risk of injury to the cat's brainstem, we attempted only 1 insertion of the medullary recording electrodes into each cat. However, when the intracortical stimulation was initiated 14–20 days later, we recorded compound neuronal action potentials in the pyramidal tracts of three animals before and after the 140 h of stimulation.

### 2.2. Prolonged electrical stimulation with the implanted microelectrodes and analysis of the microelectrode sites

To determine the correlations between the microstimulation and neuronal loss around the microelectrodes, 8 non-adjacent microelectrodes of each array's 16 microelectrodes (Fig. 1C) were stimulated for 140 h (7 h per day) over 20 consecutive days, excluding weekends and holidays. The stimulus was charge-balanced, controlled-current and cathodic phase first. It was continuous throughout the 7 h, at 50 Hz, 200 μs/phase, and at 2, 4 or 8 nC/phase for different arrays. The geometric charge densities ranged from 50 to 400 μC/cm^2^. The stimulus was delivered via a wireless backpack [19], allowing monitoring of the electrical impedance of each microelectrode while the animal could move about freely.

Ten to thirteen days after the last day of stimulation (the latter if the last day of stimulation was Friday), the cats were anesthetized with pentobarbitol and perfused with buffered saline and phosphate buffered formalin. The cortical tissue encompassing the microelectrode array site was embedded into paraffin and sectioned at 5 μm perpendicular to the microelectrode shanks. Tissue sections transecting the microelectrode sites were processed by immunohistochemistry (IHC) using an anti-NeuN monoclonal antibody (clone A60 from Sigma-Aldrich) which labels all neurons (Fig. 1D). For 6 arrays, odd-numbered tissue sections (1,3,5 …. ) were labeled with anti-NeuN, and adjacent slides (2,4,6 …) were labeled for neurons containing parvalbumin. Parvalbumin is expressed in interneurons in many brain regions. Those neurons are thought to be GABAergic, and to be inhibitory [15,16]. Due to the relatively low density of the neurons with parvalbumin labels, the neuron counts were performed only for 50–250 μm from the center of the microelectrode sites. For the parvalbumin labels and for the NeuN labels, the neuron density 50–250 μm from the site was normalized on the density of labeled neurons 250–500 μm from the center of the same site.

### 2.3. Quantification of neuron loss due to the electrical stimulation

In each photograph of a microelectrode site, the defect left by the electrode shank was labeled with a black spot (Fig. 1D) that could be recognized by the image processing software. The size and location of each labeled neuron and of the spot marking the center of the microelectrode site was determined (Global Lab Image, Data Translation, Inc), and the coordinates of each labeled neuron determined with respect the black spot. All distances cited are based on the center-to-center spacing of the electrode shanks (600 μm) in the photographs of the array sites (e.g., Fig. 1D) and the dimensions in the living brain, and thus independent of any issue shrinkage that may have occurred during tissue processing.

For quantification of neuron loss due to the electrical stimulation we must consider that loss of neurons will occur near the stimulated and the unstimulated microelectrodes, and that there may be some “positional bias” in the propensity for microelectrodes at different positions in the 16-microelectrode array (Fig. 1C) to inflict different amounts of neuron loss. Much of the damage around unstimulated microelectrodes may be due to injury during array implantation and to an inflammatory process accompanying the presence of the microelectrodes [20-22]. The development and evolution of that type of injury can be tracked by repeated monitoring of the electrodes' electrical impedance [23]. The co-ordinates of each labeled neuron within 250 μm of each microelectrode (~27,000 labeled neurons surrounding the 240 microelectrodes) were sorted to 1 of 200 annuli of 12,000 μm^2^ concentric to the microelectrode site. Each neuron was catalogued by the analysis software as being on the side of the planar microelectrode shank facing the shank's single microelectrode site, or on the opposite side of the shank.

**Fig. 2.**
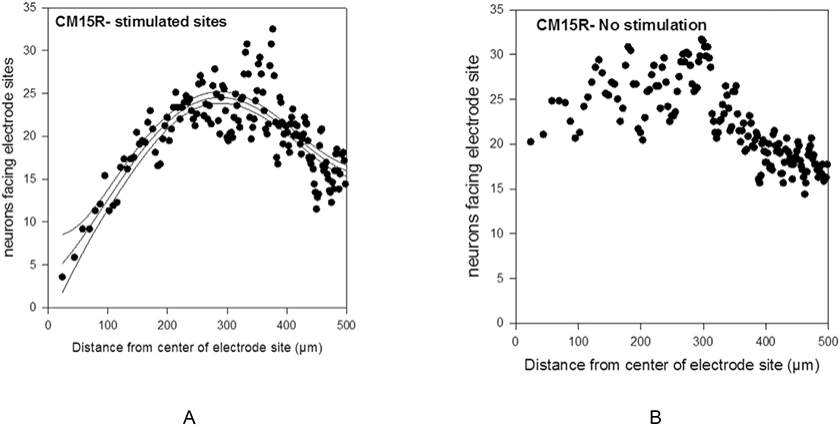
(A) Scatterplot representation of the of the number of neurons surrounding the 8 stimulated microelectrodes of intracortical array 15. (B) Number of neurons surrounding the array's 8 unstimulated sites. The ordinate of each plot is the number of labeled neurons within each 12,000 μm^2^ annulus concentric to the center of the electrode sites and on the side of the electrode facing the electrode sites. For the stimulated sites (A), the third order regression fit to the data and its 95% confidence delimiters is maximum ~270 μm from the center of the sites.

In Fig. 2A the ordinate of each dot represents the number of NeuN-labeled neurons within each constant area annulus and facing the microelectrode sites of Array 15R. Fig. 2B shows the neuron counts surrounding the array's 8 unstimulated microelectrodes. In Fig. 2A, the 3rd order regression fit to the neuron count distribution around the stimulated electrodes is maximum ~270 μm from the center of the microelectrode sites. For different arrays, these maxima ranged from 220 to 285 μm. Therefore, the loss of neurons around each stimulated and unstimulated microelectrode was quantified from the number of neurons within 250 μm and separately, within 100 μm, of the microelectrode. Those neuron counts then were normalized on the count 250–600 μm from the center of the microelectrode site, to address the expectation that the size and density of the neurons would vary, depending on the array's location and depth in the post-cruciate gyrus. The sum of the normalized counts surrounding the array's 8 stimulated microelectrode sites was normalized again on the counts around the array's 8 unstimulated sites to yield the array’ Stim/Nostim ratio (value). Separate Stim/Nostim values were calculated for the neuron counts on the side of the microelectrode shanks facing the microelectrode sites, for the neurons not facing the microelectrode sites, and for the neurons both facing and not facing the sites. These calculations of the array's Stim/Nostim are intended to average out any positional bias for the neuron loss surrounding microelectrodes at different positions in the 16-microelectrode arrays (Fig. 1C).

For each animal, the (non-parametric) Spearman's rank correlation coefficient, with provision for ties in the ranking, was calculated from the sets of normalized Stim/Nostim values from the animal's 15 arrays, using MINITAB 16 statistical software. The geometric charge density (QD) was calculated as Q/(electrode site geometric area). The array's charge per phase (Q) and geometric charge density at the surface of the electrode (QD) are the array's independent variables, and the array's Stim/Nostim ratio is the dependent variable. In separate arrays, the geometric area of the square activated iridium oxide electrode site was 2000 μm^2^ (7 arrays) or 4000 μm^2^ (8 arrays), allowing Q and QD to be varied independently.

## 3. Results

**Fig. 3.**
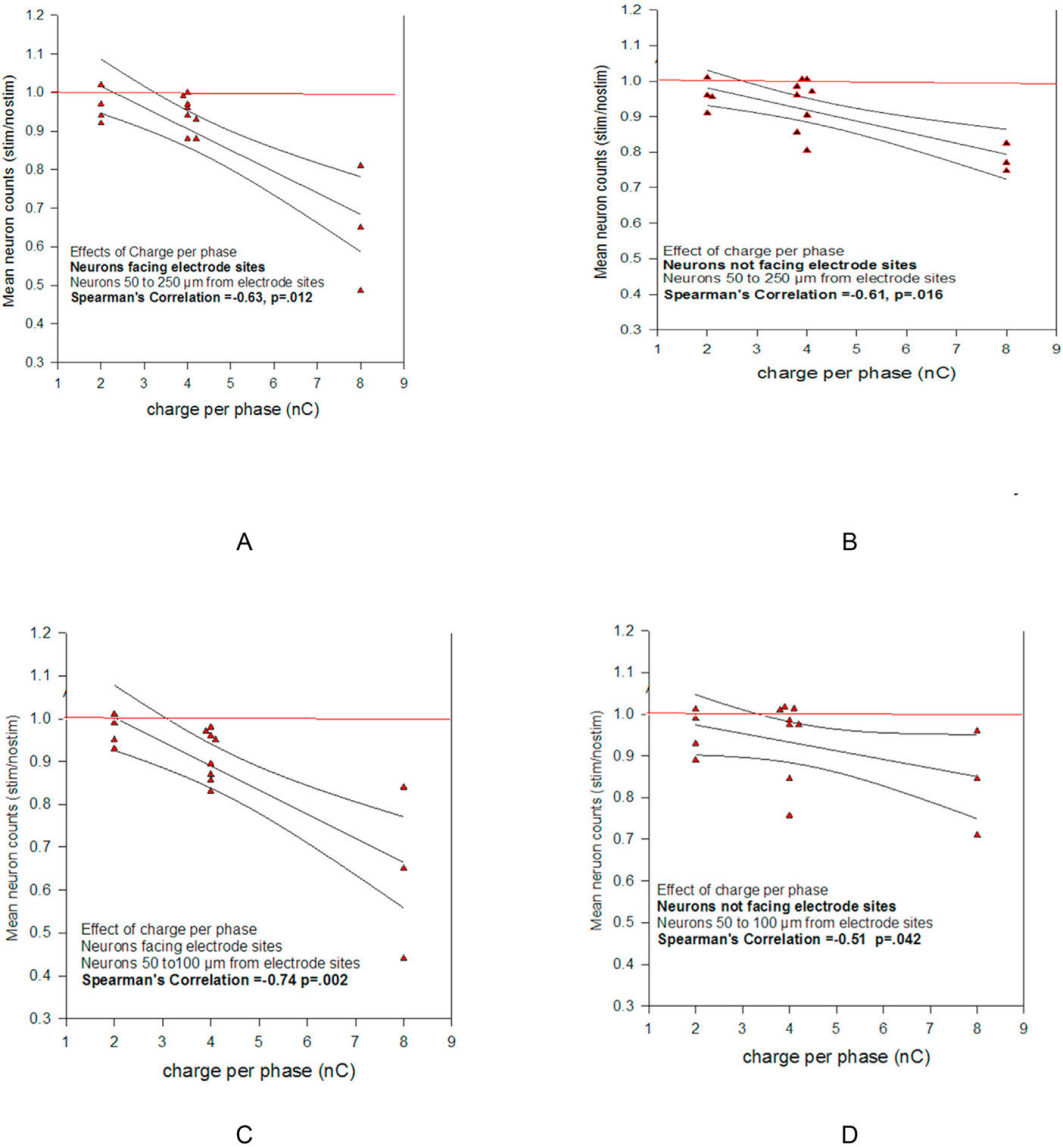
The relation between the Stim/Nostim ratio of the neuron counts for the 15 intracortical arrays. The solid horizontal line at Stim/Nostim = 1.0 represents the condition where the 140 h of stimulation at 50 Hz induced no difference in the density of neurons surrounding the stimulated vs. the non-stimulated electrode sites within the specified range of distances from the of the cerebral cortex. Smaller Stim/Nostim values signify greater loss of neurons. For different arrays, Q was 2, 4 or 8 nC for each phase of the biphasic charge-balanced stimulus. In the figures the abscissa (Q) position of some of the symbols was shifted slightly to avoid their overlap. The interaction of Q with Stim/Nostim is quantified as the non-parametric Spearman's rank correlation coefficient. The plots show the 1st order regression for the data points and the corresponding 95% confidence intervals. The strongest and weakest correlations between neuron loss and Q are seen in Panels C and D, respectively (which also are shown in Table 1 on the 4th row and 5th row, respectively).

Fig. 3 shows the relations between Stim/Nostim and the stimulus charge per phase (Q) for the neurons surrounding the microelectrodes of each of the 15 intracortical arrays. Smaller values of Stim/Nostim signifies greater loss of neurons. The instances in which the Stim/Nostim values in Fig. 3 slightly exceed 1.0 are indicative of the small uncontrolled error that is intrinsic to the analysis. Key values of the Stim/Nostim ratios shown in Fig. 3 are summarized in Tables 1 and 2. The distances listed in Column 1 of Tables 2A and 2B are with respect to the center of the microelectrode track, as depicted by the black spot in Fig. 1D. Greater absolute value of the Spearman's rank correlation coefficient indicates greater loss of neurons with increasing Q. Smaller values of Stim/Nostim signifies greater loss of neurons. The effect of distance from the electrode sites on neuron loss at specific values of Q is summarized in Table 2. At a stimulus amplitude of 8 nC/phase, the neuron loss due to the stimulation 50–250 μm from the microelectrode sites is nearly identical to the loss 50–100 μm from the sites, and only slightly less than the loss 50–400 μm from the sites. This illustrates the large radial span of the neuron loss due to the stimulation at 8 nC/phase. Some of the variance in each array's Stim/Nostim ratio at the smallest charge per phase (2 nC/phase) probably can be attributed to variance in the neuron loss around the arrays' unstimulated electrodes, as well as to variance in the contribution of the mechanically-induced loss of neurons around the stimulated electrodes. The variance in Stim/Nostim at 2 nC/phase in the brain tissue not facing electrode sites, and within 100 μm of the center of an electrode site (Fig. 3D) almost certainly is due to variance in the mechanically-induced loss of neurons.

The negative values of the Spearman's correlation coefficients shown in Fig. 3 and listed in Table 2A signify that, as expected, there was greater loss of neurons (Smaller value of Stim/Nostim) at greater Q. Over the range of 2–8 nC/phase, the strongest correlation (smallest p-values) between neuron loss and Q is on the side of the microelectrode shanks facing the microelectrode sites, and within 100 μm of the center of the shanks (4^th^ row in Table 2A and Fig. 3C). The effect of Q on neuron loss is smallest (Spearman's correlation coefficient = −0.51, p = 0.042) close to and on the side of the microelectrode shanks not facing the microelectrode sites (50–100 μm–5^th^ row in Table 2A and Fig. 3D).

**Fig. 4.**
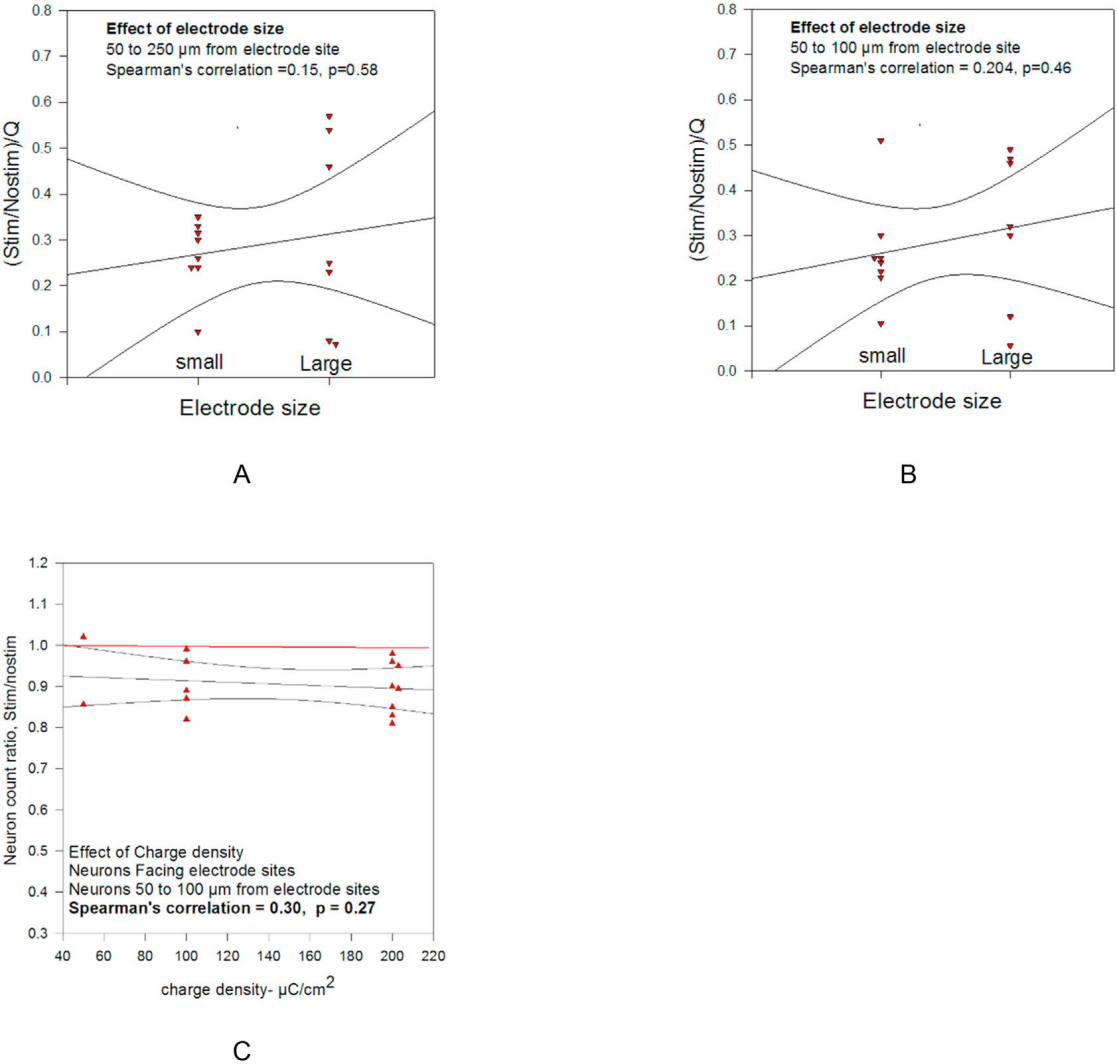
Relation between the microelectrode site size (small = 2000 μm^2^, large = 4000 μm^2^), and the normalized Stim/Nostim ratios for the 15 electrode arrays, for neurons facing the electrode sites. (A) for neurons 50 to 100 and (B) 50–250 μm from the electrode sites. (C) The correlation between the Stim/Nostim ratio vs. the geometric charge density for the 15 arrays.

The contribution of stimulus charge density (QD) to neuron loss was determined by comparing the normalized neuron loss ratio (Stim/Nostim)/Q for the 8 microelectrode arrays with 16 large microelectrode sites (4000 μm^2^) with the loss surrounding the 7 arrays with 16 small (2000 μm^2^) sites. Larger values of (Stim/Nostim)/Q signify less neuron loss per unit of Q. Fig. 4A and B shows the greater, but not statistically significant, difference in (Stim/Nostim)/Q for the large microelectrode sites. This effect of QD on neuron loss is much smaller than the effect of Q on neuron loss (Fig. 3 and Table 2).

Fig. 4C illustrates an alternative method of quantifying the effect of QD on Stim/Nostim. Here the Spearman's correlation for the 15 microelectrode arrays is positive (0.30), but not statistically significant (p = 0.27).

**Fig. 5.**
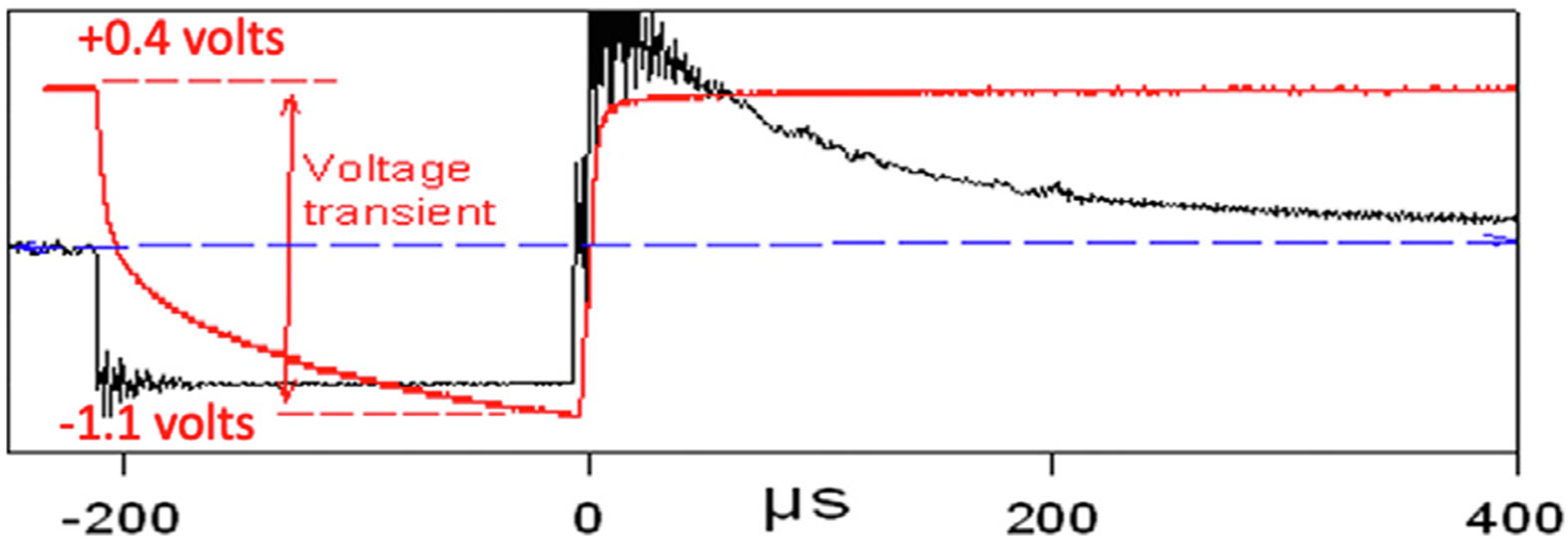
A voltage transient induced by a 200 μsec, 4 nC, 20 μA cathodic current pulse injected though a 4000 μm^2^ intracortical microelectrode.

Some of the adverse effects of greater charge density on Stim/Nostim shown in Fig. 4 may be due to incompletely reversed electrochemical of reactions during injection of the charge-balanced stimulus pulse pair. Fig. 5 shows a voltage transient of ~1.5 V that was induced by a 200 μsec, 4 nC, 20 μA cathodic current pulse injected through a 4000 μm^2^ microelectrode (Array CM4R). Table 3 lists the means and standard deviations of the voltage transients induced by current pulses injected through the 8 or 7 stimulated microelectrodes of each of the 15 intracortical microelectrode arrays, before and after the 140 h of intracortical microstimulation. We did not attempt to partition the voltage transients into ohmic and interface polarization components due to the difficulty of objectively separating those components of the voltage transients, as illustrated in Fig. 5. As expected, greater stimulus charge per phase (Column 3) induced larger voltage transients, and at a particular charge per phase the amplitude of the transients was greater for the small (2000 μm^2^) electrode sites. During the 140 h of stimulation the amplitude of the transients from each array did not change by more than 20% (Columns 5 & 6).

Previously, we reported that intracortical microstimulation may induce depression of the excitability of cortical neurons [24] so it is important to determine if depression of neuronal electrical excitability is induced by intracortical stimulation at amplitudes that do not induce detectable loss of neurons. Some neurons of the feline cruciate gyrus project into the medullary pyramidal tract, and in 8 animals recording electrodes were implanted into the tract. The exposed area of these elongated recording electrodes was moderately large (~1 mm^2^), and they recorded the averaged compound evoked responses rather than the action potentials of individual axons. In 3 of 8 animals we were able to record the compound responses evoked in the pyramidal tract by the intracortical microelectrodes before and after the 140 h of intracortical stimulation (Table 4).

**Fig. 6.**
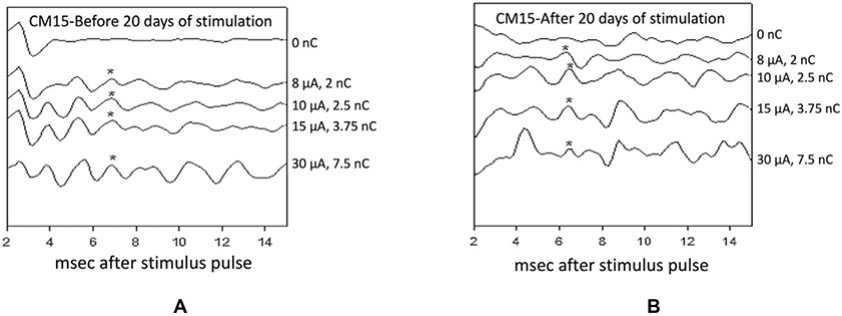
The averaged compound action potentials (ACAPs) recorded from the pyramidal tract before (A) and after (B) the 20 days (140 h) of stimulation of intracortical array CM15L at 8 microamps (2nC/phase). The compound pyramidal tract responses were evoked by stimulating with the one of the intracortical microelectrodes that had delivered the 140 h of stimulation. A compound action potential (*) with post-stimulus latency of ~6.5 msec was induced by stimuli of 8 microamps (2 nC/phase) before and also after 20 days of intracortical microstimulation. Compound responses with other post-stimulus latencies were induced by stimuli of 10 microamps or greater. 140 h of stimulation at 4 nC/phase induced a persisting elevation of the threshold of the pyramidal tract response.

Fig. 6 shows the averaged evoked responses (averaged compound action potentials, ACAPs) recorded from the pyramidal tract before and after the 20 days of intracortical stimulation of animal CM15 for which Q, the amplitude of the 140 h of stimulation through the 8 stimulated microelectrodes, was 2 nC/phase. The ACAPs were obtained by averaging the response to 1024 consecutive presentations of the intracortical stimulus. The duration of the charge-balanced stimulus pulse pairs evoking the responses was 250 μsec/phase. These ACAPs had the same post-stimulus latencies before and after the 20 days (140 h) of stimulation. The ACAP at ~6.5 msec was present before and after the 140 h of stimulation. It was not present when the stimulus amplitude was 0 nC/phase, and its amplitude was unchanged as the stimulus amplitude was increased from 2 to 10 nC/phase, demonstrating that it was not an artifact of the stimulus. In 2 other animals in which the Q of the 140 h of stimulation exceeded 2 nC per phase, the threshold of the ACAPs was elevated after the 140 h of stimulation at 50 Hz, relative to its threshold before the 140 h (Table 4). This effect of Q on neuron excitability was at least as strong as the effect of Q on neuron loss (Table 2). However, a stimulus of 2 nC/phase did induce action potentials in the pyramidal tract neurons throughout the 140 h of stimulation (Fig. 6A and B).

**Fig. 7.**
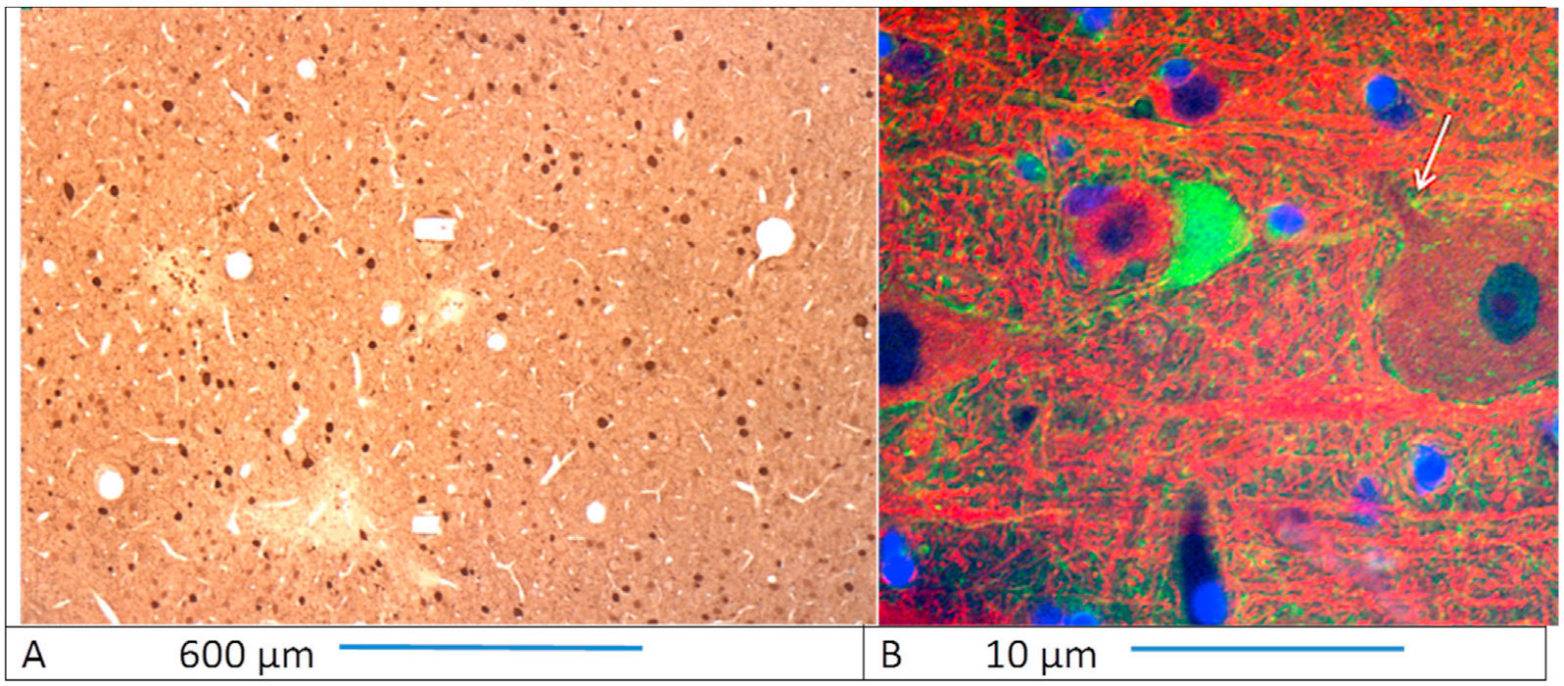
A: 5 μm section of the postcrucate gyrus of cat CM21R showing neurons immuno-labeled for parvalbumin (Small brown spots). B: A cortical neuron and its axon labeled with a green chromophore to reveal parvalbumin. Its axon (broken green line) appears to be synapsing onto the initial segment of the adjacent large neuron (white arrow), as is characteristics of the inhibitory cortical chandelier neurons [15].

In 6 animals the neurons were immuno-labeled for parvalbumin, a protein found in inhibitory neurons in the cerebral cortex [15,25], and the neurons in adjacent histology sections were labeled for NeuN [26]. Fig. 7A shows parvalbumin-labeled neurons from the post-cruciate gyrus of cat CM21R surrounding a microelectrode site. Fig. 7B shows the axon of a neuron labeled for parvalbumin synapsing onto the initial segment of a large cortical neuron, as is characteristic of these inhibitory neurons [15]. As described in METHODS, the counts of the neurons labeled for NeuN and for parvalbumin were performed on adjacent 5 μm histology sections from each microelectrode site. The number of labeled neuron 50–250 μm from the center of the microelectrode sites were normalized on the number of labeled neurons 250–500 μm from the center of the same site. Table 5 shows the Stim/Nostim ratios and the paired t-tests for the neuron counts around the stimulated and unstimulated microelectrode sites from the 6 intracortical arrays. For Arrays 21Right, 16Left and 4Left, the Stim/Nostim neuron count ratios were significantly different from 1.0 at the 0.05 level of significance for the neurons labeled for NeuN and also for the neurons labeled for parvalbumin. For arrays 19Right, 14Left and 14Right, the Stim/Nostim ratios were not significantly different from 1.0 at the 0.05 level of signifiance for the neurons labeled for NeuN or for those labeled for parvalbumin. These data suggest similar vulnerability to stimulation-induced neuron injury for the NeuN-labeled and for the parvalbumin-labeled neurons. However, at the greatest charge per phase (Array 4Left, stimulated at 8 nC/phase) the overall neuron loss due to the stimulation was greatest (Fig. 3), suggesting that the inhibitory neurons labeled for parvalbumin could be somewhat more resistant to damage by the stimulation than are some of the of subtypes of neurons that are labeled with anti-NeuN (see Discussion).

## 4. Discussion

The key findings from the study are: (1) The progressive increase in loss of cortical neurons during prolonged intracortical microstimulation across the range of stimulus amplitudes (2–8 nC/phase). (2) The relatively small role of stimulus charge density (QD), compared to charge per phase (Q), in the induction of neuron loss, (3) the similarity in the stimulation-induced neuron loss for all cortical neurons and for a major class of inhibitory critical containing parvalbumin. (4) The relatively small effect of stimulus charge density and electrode size (Fig. 4) on neuron loss, relative to the larger effect of stimulus charge per phase, suggesting that electrochemical processes at the electrode-tissue interface related to charge injection (e.g., brief but reversibly changes in pH) played a relatively small role in neuron loss. Conversely, the strong correlation between charge per phase and (the amount of) neuron loss is consistent with the role of prolonged induced neuronal activity in the neuronal damage (10). However, the small effect of stimulus charge density (and the related effect of electrode size, Fig. 4) also demonstrates the risks attendant to efforts to minimize mechanical injury by minimizing the size of penetrating stimulating microelectrodes.

As expected, the amplitude of the electrode voltage transients increased with increasing charge per phase, and the amplitude of the transients was greater for the small (2000 μm^2^) vs. the large (4000 μm^2^) electrode sites (Table 3). Fig. 4A and B do suggest a small effect of electrode site size on neuron loss. The small effect of charge density on neuron loss (Fig. 4C) could be due to incomplete reversal of electrochemical events, and the accompany pH changes accompanying stimulus charge injection (10,11). In an earlier study using implanted microwire microelectrodes [8], we showed that 7 h of intracortical microstimulation with activated iridium oxide microelectrodes at a charge of 4 nC/phase and a QD of ~200 μC/cm^2^ induced loss of neurons within 200 μm of the microelectrodes. That finding was confirmed in the present study, in Fig. 3 and Table 1. Table 1 also details the greater loss of neurons (smaller Stim/Nostim) close to the microelectrodes (50–100 μm) and on the side of the microelectrode shank facing the microelectrode site. This positional effect of neuron loss was not unexpected, but to our knowledge has not been previously reported and quantified.

In the present study, to better dissociate and define the roles of Q and QD in the induction of neuron loss, we used square microelectrode sites of two sizes (geometric surface areas of 2000 and 4000 μm^2^) and we stimulated with overlapping ranges of Q and QD and the greatest portion of the neuron loss was due to Q. If the intracortical stimulus can be delivered with minimal loss of neuron through moderately smaller electrode sites (which operate at greater QD/Q), the smaller electrode sites affixed to smaller penetrating probe shanks may induce less mechanical injury [20-22], thereby allowing greater spatial density of electrically independent electrode sites and greater spatial focusing of the microstimulation. However, we found only a small range of Q that would activate the pyramidal tract neurons (Fig. 6) but would not induce elevation of the Q needed to activate the pyramidal tract neurons (Table 4) or induce loss of cortical neurons during 140 h of microstimulation (Fig. 3 and Table 1). Also, Fig. 4 does suggest at least a weak effect of electrode site size on the loss of neurons during the stimulation. The smaller electrodes do operate at greater charge density and amplitude of their voltage transients tends to be slightly greater (Table 3). The greater amplitude of the transients suggests that electrochemical processes related to charge injection, including pH changes, may not be completely reversed after each biphasic charge-balanced stimulus pulse pair (8,10).

There remains the question of how the composition of the microelectrode sites (here activated iridium oxide) and how the site's geometric aspect ratio (here they were square) may have affected the outcome, and thus the extent to which our findings could be generalized to other electrode materials and to other electrode site configurations. A related issue is the electrical conductivity of the materials that come in contact with the tissues. The majority of multisite silicon devices leave their sidewalls and backside uninsulated, exposing silicon which is typically electrically-conductive [27]. Coating these regions with insulating material such as silicon dioxide could potentially yield a different tissue response following long-term stimulation. Absence of the insulation layer could affect both the numerator and denominator of the Stim/Nostim ratio. For the former, the electrical field could spread to the back of the probe, particularly when Q, the charge per phase is large (e.g., 8 nC/phase), and the degree to which the amplitude of charge injection affects the viability of neurons on the backside of the probe would requires further investigation. For the latter, tissue response could differ for silicon and for electrical insulators such as silicon dioxide. Finally, displacement of brain tissue by the electrode shanks and accompanying migration of brain cells could account for some of the changes in neuron density surrounding the electrode sites (19), and might affect the Stim/Nostim ratio if it differs for the stimulated and unstimulated electrodes.

We examined only a single stimulus pulse duration (200 μsec per phase of the charge-balanced pulse pair), so the role of current density (as distinct from geometric charge density) in the induction of neuronal loss was not evaluated. Other stimulus parameters, including stimulus pulse duration, may affect the ability of the stimulus to induce neurons to generate action potentials without injuring them. Other investigators [11,12] have shown that the range of stimulus amplitudes margin for avoiding electroporation of the neuron membranes was greatest when the stimulus pulse duration was close to the neuronal chronaxie (~100–250 μsec/phase for neurons in the cerebral cortex). On that basis, we selected a pulse duration of 200 μsec/phase of the biphasic cathodic-first charge-balanced stimulus.

Electrodes implanted in the medullary pyramidal tract recorded the neuronal activity induced by the electrical stimulation in the post-cruciate cerebral cortex. With 250 μsec/phase cathodic-first stimulus pulses, compound action potentials were evoked by a stimulus of 2 nC/phase (Fig. 6) at which Q the intracortical microstimulation induced minimal loss of neurons during 140 h of stimulation (Fig. 3 and Table 1). At this stimulus amplitude, the threshold stimulus for the evoked response did not change measurably during the prolonged stimulation.

It is important to determine if the parameters for minimally injurious stimulation are similar for different types of cortical neurons. To that end, neurons from 6 animals were labeled for NeuN (all neurons), and adjacent histology sections were labeled for the calcium-binding protein parvalbumin which is present in inhibitory GABAergic cortical neurons: the basket cells and chandelier cells [15,25]. If these inhibitory neurons do differ from the excitatory cortical neurons in their vulnerability to injury by the stimulation, the excitatory/inhibitory (E/I) balance in the cerebral cortex could be altered [13]. For example, reduction of the density of inhibitory cortical neurons has been observed in the CNS of persons with schizophrenia [13,14]. Animal models with schizophrenia-like phenotypes had fewer parvalbumin interneurons in their hippocampus [15,16]. Table 5 shows that, for the range of Q evaluated, the vulnerability to stimulation-induced neuronal injury was similar for the neurons labeled for NeuN and those labeled for parvalbumin (similar values of Stim/Nostim). However, Table 5 does hint that the vulnerability of neurons to damage by high amplitude stimulation (e.g., Array 4Left, stimulated with 8 nC/phase) may differ for different types of neurons in the cerebral cortex. That matter may be worthy of further study using a larger sample of microelectrodes and marker for different types of neurons.

## Figures and Tables

**Table 1:** Effect of position relative to electrode sites on Stim/Nostim for neurons surrounding the electrode sites, as plotted in Fig. 3.

Array #	Q, nC per phase(*)	Stim/Nostim Neurons 50 to 100 μm from electrode shank			Stim/Nostim Neurons 50 to 250 μm from electrode shank		
	
		F	NF	FNF	F	NF	FNF
1	2	1.02	1.01	1.03	1.01	0.96	1.01
2	2	0.95	0.89	0.91	0.92	1.01	0.99
3	2	0.99	0.99	0.93	0.97	0.96	0.98
4	2	0.93	0.93	1.01	0.94	0.91	0.94
5	4	0.98	0.94	0.84	0.98	0.97	0.99
6	4	0.83	1.01	1.02	0.88	0.86	0.88
7	4	0.89	1.01	0.99	0.99	0.86	0.99
8	4	0.95	0.97	0.94	0.96	0.99	0.99
9	4	0.87	0.76	0.84	0.93	0.96	0.85
10	4	0.86	0.98	0.79	0.89	0.99	0.99
11	4	0.97	0.97	0.98	0.99	0.81	1.03
12	4	0.94	1.01	0.96	0.94	0.98	0.93
13	8	0.65	0.84	0.65	0.65	0.77	0.75
14	8	0.84	0.96	0.84	0.81	0.82	0.84
15	8	0.44	0.71	0.74	0.49	0.74	0.68

F: Neurons counted on the side of the electrode shank facing the electrodes site. NF: Neurons counted on the side of the electrode shank not facing electrodes site. FNF: Neurons counted on the sides of the electrode shank facing & not facing electrode site.

**Table 2A:** Effect of stimulus charge per phase (Q), distance from the microelectrode sites, and position in the tissue relative to the microelectrode sites (Facing or not facing the sites) on neuron counts near the intracortical microelectrodes after 140 h of microsimulation. Larger Spearman's correlation signifies greater loss of neurons due to the stimulation. Radial span of neuron counts (*) is with respect to the center of the microelectrode site.

Where neurons counted (μm from center of the electrode site)	Where neurons counted	Span of stimulus Q (nC/ phase)	Spearman's correlation for Stim/Nostim after 140 h of stimulation at 50 Hz
50–250 μm	Facing electrode sites	2–8 nC	−0.63 p = 0.012
50–250 μm	Not facing sites	2–8 nC	−0.61 p = 0.016
50–250 μm	Facing & Not facing sites	2–8 nC	−0.65 p = 0.009
50–100 μm	Facing sites	2–8 nC	−0.74 p = 0.002
50–100 μm	Not facing sites	2–8 nC	−0.51 p = 0.042
50–100 μm	Facing & Not facing sites	2–8 nC	−0.53 p = 0.038

**Table 2B:** Neuron Loss (Stim/Nostim ratio) at various distances from the stimulating microelectrodes, after 140 h of stimulation at 4 nC/phase. Neuron counts are from the side of electrode shank facing the electrode site. Smaller values of Stim/Nostim signifies greater loss of neurons due to the stimulation.

Where neurons counted (μm from center of electrode site)*	Stimulus amplitude	Stim/Nostim ratio after 140 h of stimulation at 4 nC/phase and at 50 Hz
50–100 μm	4 nC	0.91±0.058
50–250 μm	4 nC	0.94±0.050
50–400 μm	4 nC	0.96±0.056
50–100 μm	8 nC	0.64±0.20
50–250 μm	8 nC	0.65±0.16
50–400 μm	8 nC	0.76±0.08

**Table 3:** Effect of stimulus charge per phase and electrode site size on the amplitude of electrode voltage transients.

Cat & Array (Left(L) or right (R)	Array #	Stimulus (nC/ phase & μA	Electrode size	Transient volts Before 140 h of Stimulation (Mean & SD for the array's 7 or 8 stimulated electrodes)	Transient volts After 140 h of stimulation (Mean & SD for the array's 7 or 8 stimulated electrodes)
CM11(L)	1	2 nC, 10 μA	2000 μm^2^	1.23±0.11	1.36±0.46
CM19(R)	2	2 nC, 10 μA	2000 μm^2^	1.22±0.31	1.21±0.40
CM11(R)	3	2 nC, 10 μA	4000 μm^2^	1.15±0.11	1.11±0.39
CM21(R)	4	2 nC, 10 μA	4000 μm^2^	1.20±0.21	1.20±0.46
CM2(R)	5	4 nC, 20 μA	2000 μm^2^	1.54±0.31	1.56±0.35
CM6(L)	6	4 nC, 20 μA	2000 μm^2^	1.60±0.21	1.42±0.26
CM10(L)	7	4 nC, 20 μA	2000 μm^2^	1.50±0.27	1.43±0.39
CM20(R)	8	4 nC, 20 μA	2000 μm^2^	1.54±0.31	1.56±0.35
CM4R	9	4 nC, 20 μA	4000 μm^2^	1.35±0.36	1.31±0.40
CM4L(L)	10	4 nC, 20 μA	4000 μm^2^	1.32±0.29	1.37±0.40
CM5R	11	4 nC, 20 μA	4000 μm^2^	1.40±0.31	1.42±0.38
CM2(L)	12	4 nC, 20 μA	4000 μm^2^	1.31±0.27	1.31±0.26
CM8(R)	13	8 nC, 40 μA	2000 μm^2^	1.59±0.31	1.61±0.35
CM4(L)	14	8 nC, 40 μA	4000 μm^2^	1.57±0.31	1.59±0.35
CM8(L)	15	8 nC, 40 μA	4000 μm^2^	1.54±0.31	1.58±0.35

**Table 4:** Effect of charge per phase (Q) during prolonged intracortical stimulation on the responses of pyramidal tract neurons near the intracortical microelectrodes.

Array	Q during 140 h of stimulation	Threshold of pyramidal tract response before 140 h of stimulation	Threshold of pyramidal tract response after 140 h of stimulation
CM15L	2 nC	<8 μA, 2 nC	<8 μA, 2 nC
CM6R	4 nC	<8 μA, 2 nC	>15 μA
CM4L	8 nC	<8 μA, 2 nC	>15 μA

**Table 5:** Stimulation-induced loss of cortical neurons (Stim/Nostim) from 6 intracortical microelectrode arrays, for neurons labeled for NeuN and for parvalbumin.

Array	Q, nC/phase during 140 h of stimulation	Neuron count ratio (Stim/Nostim) -For NeuN (Facing & not facing electrode sites, & 50–250 μm from center of site)	Neuron count ratio (Stim/Nostim) -For Parvalbumin (Facing & not facing electrode sites, & 50–250 μm from center of site)
21Right	2	0.94 p = 0.011 *	0.93 p = 0.003 *
19Right	2	0.98 p = 0.11	1.07 p = 0.49
14Left	4	0.90 p = 0.93	0.94 p = 0.28
16Left	4	0.90 p = 0.01 *	0.79 p = 0.037 *
14Right	4	0.93 p = 0.070	0.98 p = 0.073
4Left	8	0.48 p = 0.000 *	0.81 p = 0.028 *

(*) signifies significant Stim/Nostim neuron count ratio (p < 0.5) after 140 h of stimulation.
